# Putative second hit rare genetic variants in families with seemingly GBA-associated Parkinson’s disease

**DOI:** 10.1038/s41525-020-00163-8

**Published:** 2021-01-05

**Authors:** Muhammad Aslam, Nirosiya Kandasamy, Anwar Ullah, Nagarajan Paramasivam, Mehmet Ali Öztürk, Saima Naureen, Abida Arshad, Mazhar Badshah, Kafaitullah Khan, Muhammad Wajid, Rashda Abbasi, Muhammad Ilyas, Roland Eils, Matthias Schlesner, Rebecca C. Wade, Nafees Ahmad, Jakob von Engelhardt

**Affiliations:** 1 Institute of Pathophysiology, University Medical Center of the Johannes Gutenberg University Mainz, Mainz, Germany; 2 Institute of Biomedical and Genetic Engineering (IBGE), Islamabad, Pakistan; 3 Department of Biochemistry, Quaid-i-Azam University, Islamabad, Pakistan; 4 Heidelberg Center for Personalized Oncology (DKFZ-HIPO), German Cancer Research Center (DKFZ), Heidelberg, Germany; 5 Molecular and Cellular Modeling Group, Heidelberg Institute of Theoretical Studies (HITS), Heidelberg, Germany; 6 The Signalling Research Centres BIOSS and CIBSS, University of Freiburg, 79104 Freiburg, Germany; 7 Department of Zoology, PMAS-Arid Agriculture University, Rawalpindi, Pakistan; 8 Department of Neurology, Shaheed Zulfiqar Ali Bhutto Medical University, Islamabad, Pakistan; 9 Department of Microbiology, University of Balochistan, Quetta, Pakistan; 10 Department of Biological Sciences, University of Okara, Okara, Pakistan; 11 Faculty of Mechanical Engineering, GIK Institute of Engineering Sciences and Technology, Topi, 23460 Pakistan; 12 Center for Digital Health, Berlin Institute of Health and Charité Universitätsmedizin Berlin, Berlin, Germany; 13 Health Data Science Unit, Bioquant, Medical Faculty, University of Heidelberg, Heidelberg, Germany; 14 Bioinformatics and Omics Data Analytics, German Cancer Research Center (DKFZ), Heidelberg, Germany; 15 Center for Molecular Biology of the University of Heidelberg (ZMBH), DKFZ-ZMBH Alliance, and Interdisciplinary Center for Scientific Computing (IWR), Heidelberg, Germany

**Keywords:** Genetics research, Parkinson's disease

## Abstract

Rare variants in the beta-glucocerebrosidase gene (*GBA1*) are common genetic risk factors for alpha synucleinopathy, which often manifests clinically as GBA-associated Parkinson’s disease (GBA-PD). Clinically, GBA-PD closely mimics idiopathic PD, but it may present at a younger age and often aggregates in families. Most carriers of GBA variants are, however, asymptomatic. Moreover, symptomatic PD patients without GBA variant have been reported in families with seemingly GBA-PD. These observations obscure the link between GBA variants and PD pathogenesis and point towards a role for unidentified additional genetic and/or environmental risk factors or second hits in GBA-PD. In this study, we explored whether rare genetic variants may be additional risk factors for PD in two families segregating the PD-associated *GBA1* variants c.115+1G>A (ClinVar ID: 93445) and p.L444P (ClinVar ID: 4288). Our analysis identified rare genetic variants of the HSP70 co-chaperone DnaJ homolog subfamily B member 6 (DNAJB6) and lysosomal protein prosaposin (PSAP) as additional factors possibly influencing PD risk in the two families. In comparison to the wild-type proteins, variant DNAJB6 and PSAP proteins show altered functions in the context of cellular alpha-synuclein homeostasis when expressed in reporter cells. Furthermore, the segregation pattern of the rare variants in the genes encoding DNAJB6 and PSAP indicated a possible association with PD in the respective families. The occurrence of second hits or additional PD cosegregating rare variants has important implications for genetic counseling in PD families with *GBA1* variant carriers and for the selection of PD patients for GBA targeted treatments.

## Introduction

Rare variants in the gene *GBA1*, which encodes beta-glucocerebrosidase (GBA, OMIM: #606463; EC 3.2.1.45), are considered as risk factors for developing alpha synucleinopathy manifesting clinically as Parkinson’s disease (PD)^1,2^. The GBA-associated PD (GBA-PD) often aggregates in families but the connection between *GBA1* variants and PD pathogenesis remains ambiguous. Contributing to this ambiguity is an unknown molecular mechanism by which GBA variants translate into cellular alpha-synuclein mishandling. Genetic observations such as the occurrence of non-symptomatic *GBA1* variant carriers (e.g. incomplete penetrance) and a high rate of PD symptomatic non-carriers (e.g. phenocopies of mostly unknown origin) in seemingly GBA-PD families further obscure the link between GBA and PD^3–5^. From a genetic point of view, reduced penetrance and phenocopies in GBA-PD families can be explained by interactions between *GBA1* variants and unidentified additional genetic and/or environmental risk factors or second hits. For instance, additional risk factors may influence cellular alpha-synuclein homeostasis concurrently with *GBA1* variants in an epistatic manner, thereby modifying the risk of alpha synucleinopathy attributed to GBA variants and hence their penetrance. Alternatively, additional risk factors may act independently of GBA variants and increase the PD risk non-epistatically of the *GBA1* locus, thereby resulting in the occurrence of phenocopies in GBA-PD families.

The identification of additional factors such as second genetic hits can improve the clinical management of GBA-PD on many fronts. For instance, this knowledge will enable genetic risk management, counseling, and clinical surveillance in seemingly GBA-PD families, which is otherwise difficult to achieve solely based on *GBA1* variants. Furthermore, GBA has recently become a prominent target for therapeutic development. Drugs such as GZ/SAR402671 (ClinicalTrials.gov Identifier: NCT02906020) and Ambroxol (ClinicalTrials.gov Identifier: NCT02941822 and NCT02914366) have been promoted for randomized interventional trials in PD patients with *GBA1* variants. These drugs aim to augment lysosomal transport and function in *GBA1* variant carriers thereby restoring healthy lysosomal function, which is required for cellular alpha-synuclein handling^6–8^. A major concern is that additional risk factors in *GBA1* variant carriers may affect cellular alpha-synuclein homeostasis and thereby may confound the outcome of these trials.

Massively parallel sequencing, when combined with familial genetic studies, has the potential to reveal high-risk rare genetic variation in complex neurodegenerative disorders that show a substantially heritable component^9^. Application of sequencing technologies in PD genetics has recently led to the discovery of additional potentially pathogenic rare genetic variation in PD patients carrying primary pathogenic mutations, thus providing evidence for oligogenic inheritance and pathway interactions in PD^10,11^. In this study, we uncovered rare variants in *DNAJB6* and *PSAP* as putative second hits in two PD families segregating *GBA1* mutations c.115+1G>A and p.L444P, respectively.

## Results

### Clinical findings

**Fig. 1:**
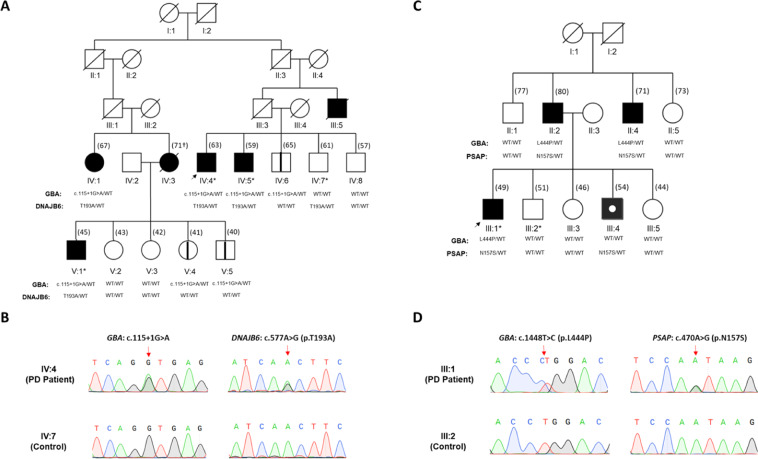
Genetic and clinical characteristics of family A and B. **A** The pedigree structure of families A and segregation of GBA: c.115+1G>A and DNAJB6; p.T193A (c.A577G) are shown. Affected individuals are shown as filled symbols and the arrow points to the index patient. The affection status of the family members of generation I and II could not be ascertained. The affection status of deceased individuals (diagonal lines) was reported by immediate family members and was confirmed by available medical records. Symbols with black lines (IV:6, V:4, and V:5) indicate asymptomatic GBA variant carriers. Asterisks mark the individuals for whom whole-genome sequencing and rare variant analysis was performed. **B** Representative Sanger sequence chromatograms showing *GBA1*: c.115+1G>A and DNAJB6; p.T193A (c.A577G) variant positions in subjects IV:4 and IV:7 of family A. Arrowhead points to heterozygous substitutions. **C** The pedigree structure of families B and segregation of GBA; p.L444P (c.1448T>C) and PSAP; p.N157S (c.A470G) variants is shown. Affected individuals are shown as filled symbols and the arrow points to the index patients. The symbol with a white circle indicates an individual with PD but without GBA variant (phenocopy PD patient, III:4). The PSAP; p.N157S (c.A470G) variant was found in all PD, and in addition in one healthy family member (IV:7). Asterisks mark the individuals for whom whole-genome sequencing and rare variant analysis was performed. **D** Representative sanger sequence chromatograms showing GBA; p.L444P (c.1448T>C) and PSAP; p.N157S (c.A470G) variant positions in subjects III:1 and III:2 of family B. Arrowhead points to heterozygous substitutions.

Family A comprised four PD subjects, with a mean age of 58.5 years (range 45–67), mean age at onset of 46.5 years (range 40–51), and a mean disease duration of 12.5 years. Depression was noted in all PD patients of the family A. Patients with longer disease duration presented with cognitive decline (Supplementary Table 1 and Fig. 1, subjects IV:1 and IV:4). The four PD patients in family A were treated with dopaminergic medication with a good response. Two of the deceased individuals (Fig. 1A: III:5 and IV:3) of family A were described to have PD. Family B comprised four male PD subjects, with a mean age of 63.5 years (range 49–80), mean age at onset of 51.5 years (range 42–59), and a mean disease duration of 13 years (Supplementary Table 1 and Fig. 1C). Subjects II:4 and III:1 in family B presented with severe depression (Supplementary Table 1). The PD patients in family B were treated with carbidopa–levodopa (Sinemet) with a good response.

### Identification and familial segregation of rare genetic variants in family A and B

The contribution of rare genetic variation to PD risk in family A and B was explored by whole-genome sequencing followed by rare variant filtering and segregation analysis. The summary of study design and data analysis is shown in Supplementary Fig. 1. Genome sequencing was performed for three PD patients in family A and one PD patient in family B (Fig. 1A, C; asterisks). Sequencing was also performed for one control individual from each family (Fig. 1A, C; asterisks), which helped to reduce the final list of candidate rare variants. The list of candidate rare variants identified in the two families is shown in Table 1.

**Table 1:** Candidate rare variants identified through genome sequencing and analysis of family A and B.

Family	Gene	OMIM_ID	Chr.	Position	Variant ID	MAF^a^	Annotation	DNA change	Protein change	ClinVAR ID	CADD_PHRED	Primers	Segregation^b^
A	*GBA*	606463	1	155210420	rs104886460	<0.0001	Splicing	c.115+1G>A	—	93445	25	Hs00280399_CE	Yes
A	*TNR*	601995	1	175372714	rs61731112	0.0034	Exonic	c.538A>C	p.N180H	224863	24	Hs00820434_CE	No
A	*CDYL*	603778	6	4716116	rs141268596	<0.0001	Splicing	c.103+1G>A	—	—	22	Hs00271238_CE	No
A	*TMEM209*	.	7	129825030	rs118139998	0.0042	Splicing	c.951+2T>C	—	—	25	Hs00372199_CE	No
A	*DNAJB6*	611332	7	157177659	rs770053224	<0.0001	Exonic	c.577A>G	p.T193A	—	22	Hs00292897_CE	Yes
B	*SUMF2*	607940	1	56144560	rs201655158	<0.0001	Exonic	c.578C>G	p.P209R	—	23	Hs00493950_CE	No
B	*GBA*	606463	1	155205043	rs421016	0.0006	Exonic	c.1448T>C	p.L483P	4288	24	See “Methods”	No
B	*MAP4*	157132	3	47912353	rs763834865	<0.0001	Exonic	c.2809T>A	p.S937T	—	28	Hs00231308_CE	No
B	*MYLK*	600922	3	123427717	rs138172035	<0.0001	Exonic	c.1440G>C	p.W656C	342893	26	Hs00238440_CE	No
B	*PSAP*	176801	10	73588740	rs756379007	<0.0001	Exonic	c.470A>G	p.N157S	—	25	Hs00436128_CE	Yes
B	*EIF4G2*	602325	11	10825847	rs772343866	<0.0001	Exonic	c.470A>C	p.Q157P	—	23	Hs00413940_CE	No
B	*NAV2*	607026	11	20136337	rs778848036	<0.0001	Exonic	c.6968C>G	p.P2390L	—	34	Hs00392827_CE	No
B	*ACACB*	601557	12	109683468	rs545762685	<0.0001	Exonic	c.5216C>G	p.P1739R	—	24	Hs00127284_CE	No
B	*GIT2*	608564	12	110390977	rs79037701	<0.0001	Exonic	c.1168G>A	p.D390N	—	33	Hs00127511_CE	No
B	*FKBP10*	607063	17	39977280	rs781812058	<0.0001	Exonic	c.1336A>G	p.M446I	—	33	Hs00405643_CE	No
B	*ABCA7*	605414	19	1052249	Novel	<0.0001	Exonic	c.C3184T	p.Q1062X	—	36	See “Methods”	No
B	*CDC25B*	116949	20	3785591	rs369357260	<0.0001	Exonic	c.1534C>A	p.R576W	—	34	Hs00211078_CE	No

^a^Minor allele frequency.

^b^Yes: all PD patients and one or more control individuals identified as heterozygous carriers. No: absent in one or more PD patients.

A *GBA1* splice-site variant (rs104886460-G/A; c.115+1G>A; ClinVar ID: 93445) was identified in the WGS data of family A. Segregation testing of this variant in family A revealed four PD subjects as heterozygous carriers of c.115+1G>A and in addition three asymptomatic carriers (Fig. 1A, symbols marked with thick lines; IV:6, V:4, and V:5). The younger asymptomatic carriers V:4 and V:5 (aged 41 and 40 years, respectively) may still develop PD or may already exhibit prodromal features of the disease as suggested previously^12,13^. We therefore assessed the three asymptomatic *GBA1*; c.115+1G>A carriers for olfactory (odor identification ability), visual and cognitive functions (Mini-Mental State Examination score), and depression (HAM-D score), which were all normal (data not shown). The occurrence of an asymptomatic carrier (IV:6, age 65 years) considerably older than the average age of PD onset in this family (i.e 46.5 years) suggests that the PD penetrance of the heterozygous variant *GBA1*; c.115+1G>A in family A is incomplete.

**Fig. 2:**
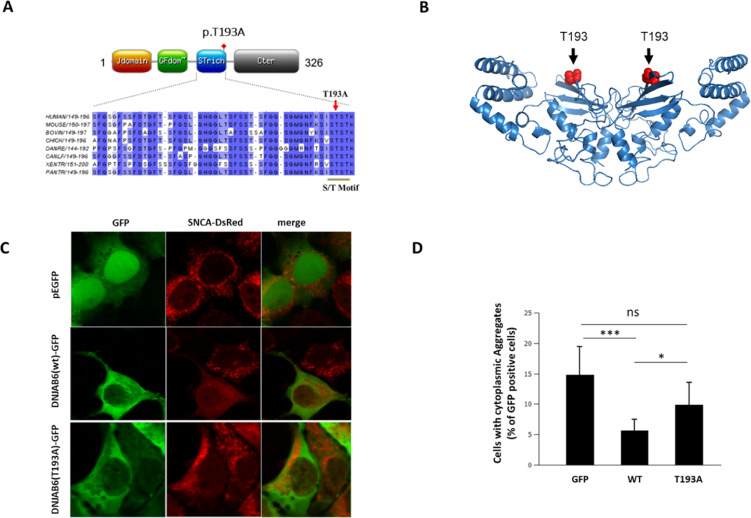
Functional impact of the PD cosegregating DNAJB6 variant c.A577G:p.T193A identified in family A. **A** Schematic diagram of the human DNAJB6 domain structure is shown. The approximate position of p.T193A is indicated. Alignment of a region of the human DNAJB6 protein sequence containing an S/T-rich motif with the corresponding polypeptide sequence from various species is shown below. Numbers indicate amino acid positions. Blue shading indicates the conservation status of the corresponding residues. Arrow points to the variant position, the S/T motif (SxSTST) is also highlighted. **B** The model structure of the DNAJB6 homodimer with the variant position (T193, red spheres) is shown. **C** Representative images showing the cellular distribution of alpha-synuclein-dsRed fusion protein in an alpha-synuclein aggregation reporter HEK293 cell line lacking endogenous DNAJB6 expression. Cells were transfected with plasmids expressing EGFP alone or as a fusion protein with wild type or the T193A variant containing DNAJB6. Focal and diffuse cytoplasmic distributions of alpha-synuclein-dsRed can be observed. **D** Quantification of focal cytoplasmic distribution of aggregated alpha-synuclein-dsRed (*n* = 3 transfections). Statistical analysis was performed by ANOVA. ***P < 0.001, *P < 0.05. Graphs represent mean and standard deviation.

Another *GBA1* gene variant (rs421016-T/C; c.1448T>C which encodes p.L444P, also called p.L483P; ClinVar ID: 4288), was identified by WGS of a PD patient (Fig. 1C, subject III:1) from family B. Segregation analysis of the p.L444P variant in family B revealed two additional PD patients as heterozygous carriers (Fig. 1C, subjects II:2 and II:4). No asymptomatic carrier of p.L444P was observed in this family. The PD patient III:4 in family B (Fig. 2C, male, black symbol with a white circle) did not segregate the GBA; p.L444P variant. He presented with action tremor, slight rigidity, and slowness of the movement in his upper right limb at the age of 49, which affected his daily activities, including writing, dressing, and his job as a restaurant worker. His motor symptoms worsened over a span of 2 years. Based on his medical history, a review of his signs and symptoms, and a neurological and physical examination he was formally diagnosed with PD at the age of 51 years (Supplementary Table 1). Due to his non-carrier status for the p.L444P variant in *GBA1*, which otherwise segregated with PD in family B, the patient III:4 was considered a phenocopy PD patient of unknown origin.

In addition to the *GBA1* splice variant c.115+1G>A segregating in family A, five single-nucleotide variants (SNVs) comprising two splice-site variants (Table 1: *CDYL* and *TMEM209* genes) and two exonic variants (Table 1: *DNAJB6* and *TNR* genes) were identified as candidates. In family B, which segregated GBA; p.L444P, the list of additional candidates comprised 11 exonic SNVs (Table 1: *SUMF2, MAP4, MYLK, PSAP, ELF4G2, NAV2, ACACB, GIT2, FKBP10, ABCA7*, and *CDC25B* genes). Besides a recently reported variant in the Tenascin-R gene (*TNR*) (rs61731112-A/C; c.A538C encoding p.N180H, ClinVAR ID 224863), which partially segregated with PD in family A, the list of additional candidates did not include exonic SNVs, CNVs, or small indels in known familial PD genes (*SNCA, PKRN, PINK1, DJ1, LRRK2, ATP13A2, PLA2G6, FBX07, VPS35, DNAJC6, SYNJ1, DNAJC13, RAB39B*)^14^. All additional candidate rare variants passed validation by bidirectional Sanger sequencing.

Exonic Rare variants c.A577G (p.T193A) in the *DNAJB6* gene (NM_058246.4, rs770053224_A/G) and c.A470G (p.N157S) in the *PSAP* gene (NM_002778.4, rs756379007_T/C) segregated with PD in family A and B, respectively. All four PD patients (IV:1, IV:4, IV:5, and V:1) of family A were heterozygous carriers for the DNAJB6; p.T193A and *GBA1*; c.115+1G>A variants whereas the three non-penetrant carriers of *GBA1*; c.115+1G>A in family A (IV:6, V:4, and V:5) lacked this concurrence. This indicates that the DNAJB6; p.T193A variant contributes to PD risk in this family (Fig. 1A). In family B, segregation of PSAP; p.N157S in all four PD patients including the phenocopy PD patient III:4 (Fig. 1C, black symbol with a white circle) indicated that the rare genetic variation in the *PSAP* gene is an independent risk factor for PD in this family which complies with a previous report^10^.

The PD cosegregating variants in *DNAJB6* and *PSAP* genes identified in this study are listed with extremely low frequencies (MAF < 0.0001) in the ethnically matched controls in GnomAD v2.1.1 (ref. ^15^) and were classified as the 1% most deleterious substitutions in the genome (CADD > 20)^16^ (Table 1). No other additional candidate rare variant segregated completely with PD in the two families.

### Functional impact of the rare variant in DNAJB6

DNAJB6, a member of the HSP40 family of J-protein co-chaperones, acts as an aggregation suppressor preventing fibrillation of several aggregation-prone proteins^17,18^. Steady-state levels and cellular distribution profiles of the wild-type DNAJB6 and the p.T193A variant proteins expressed in HEK293 cells were not significantly different (data not shown). DNAJB6 dimer-/oligomerization was also not affected by p.T193A substitution (Supplementary Fig. 2), suggesting that p.T193A substitution exerts its influence by altering the cellular function of DNAJB6 protein. The p.T193A substitution in DNAJB6 protein identified in the current study is located in a highly conserved serine/threonine-rich motif (i.e SxSTST at residues 190 and 192–195), which lines the concave side of the DNAJB6 dimer near the peptide-binding cleft (Fig. 2A, B). This structural conformation is necessary for cellular functions of the DNAJB6 protein^19,20^, which includes alpha-synuclein homeostasis^21^. We expressed wild-type DNAJB6 or p.T193A variant containing DNAJB6 proteins in an alpha-synuclein reporter cell line lacking endogenous DNAJB6 (see “Methods”). Diffuse cytoplasmic distribution of alpha-synuclein-dsRed conjugate was observed in the majority of cells in case of wild-type DNAJB6 protein expression whereas the expression of DNAJB6; p.T193A only partially restored the diffuse cytoplasmic alpha-synuclein distribution. A significantly higher number of cells expressing DNAJB6; p.T193A variant displayed a focal cytoplasmic distribution of alpha-synuclein-dsRed compared to wild-type DNAJB6. Collectively, these data indicate altered cellular homeostasis of alpha-synuclein as a direct consequence of p.T193A variant expression in HEK293 cells (Fig. 2C, D).

### Functional impact of the rare variant in PSAP

**Fig. 3:**
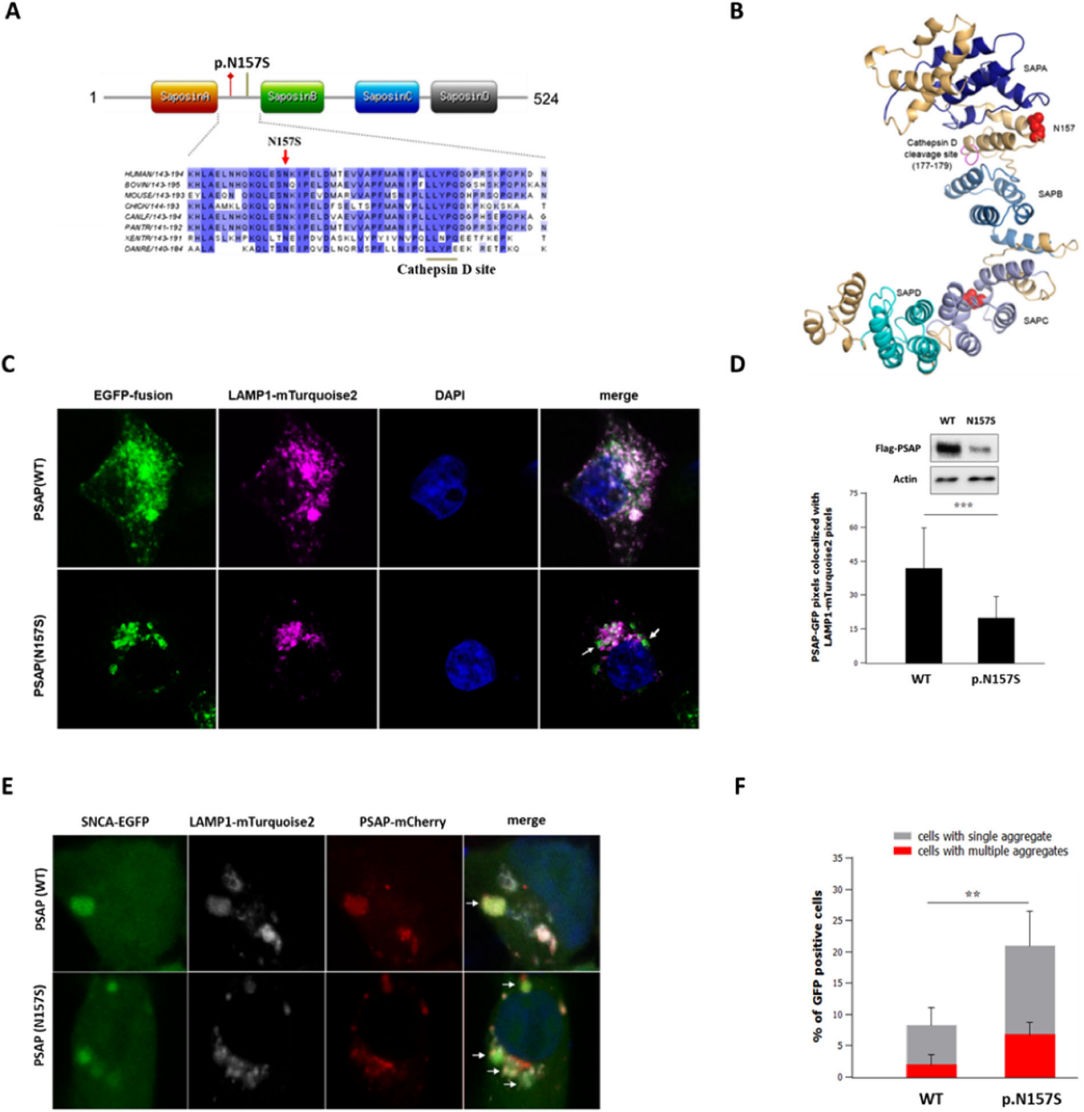
Functional impact of the PD cosegregating PSAP variant c.A470G:p.N157S identified in family B. **A** The domain structure of human the PSAP protein is shown. The location of the p.N157S variant (red) and the predicted Cathepsin D site (L177–L179) in the intersaposin A–B region is indicated (green). The alignment of human PSAP with various species is shown. Numbers indicate the position of amino acid residues. Blue shading indicates the conservation status of the corresponding residues; arrow points to the variant position. The predicted Cathepsin D site, L177–L179, is indicated (underlined in green). **B** The structure of the human PSAP protein is shown. The variant positions (N157) in the intersaposin A–B region is marked in red and the predicted Cathepsin D site (L177–L179) is displayed in pink. **C** Representative images showing the distribution of LAMP1-mTurquoise2 and PSAP-EGFP fusion proteins in HEK293T cells. DAPI was used as a nuclear stain (blue). Arrowheads in the bottom right image point to the EGFP-PSAP puncta in the perinuclear region not overlapping with LAMP1-mTurquoise2. **D** Quantification of green (PSAP-EGFP fusion protein) signal intensity overlapping with turquoise signal (mTurquoise2-LAMP1 marking the perinuclear lysosomal compartment) is shown. Graphs represent mean and standard deviation ***P = 0.001. Also shown is the immunoblot image of wild type and N157S variant containing PSAP proteins from HEK293 cells (top panel). HEK293 cells were transfected with an equal amount of plasmids expressing the wild type or N157S variant PSAP proteins as Flag-tag conjugate. Full-length blots are presented in Supplementary Fig. 3C. All blots are derived from the same experiment and gels/blots were processed in parallel. **E** Representative images showing LAMP1-positive intracellular inclusions decorated with alpha-synuclein-EGFP. Cells were transfected with three plasmids: a plasmid-expressing EGFP-tagged aggregation-prone alpha-synuclein protein (see “Methods”), a plasmid-expressing mTurquoise2-LAMP1 expression protein as a lysosomal marker, and a plasmid-expressing wild type or p.PN157S variant containing PSAP. Arrowheads in the merged panel point to the EGFP-PSAP puncta in the perinuclear region overlapping with LAMP1-mTurquoise2 as well as alpha-syuclein-EGFP puncta indicating lysosomal aggregation. **F** The quantification of lysosomal alpha-synuclein-EGFP aggregates in transfected cells is shown (*n* = 3 transfections). Statistical analysis was performed by ANOVA. Graphs represent mean and standard deviation, **P < 0.01.

Prosaposin (PSAP) is a lysosomal protein, which in its native form or processed peptides (e.g. Saposins A–D and several prosaptides) plays distinct cellular roles^22^. The PD cosegregating PSAP variant p.N157S identified in family B (Fig. 2C, D) is located in the Intersaposin A–B region of the PSAP protein (Fig. 3A). Rare potentially pathogenic variants in other regions of PSAP protein have previously been reported to increase PD risk in a GBA-dependent manner^10,23–25^. To determine the functional impact of the p.N157S variant, we expressed wild type or p.N157S variant PSAP as Flag or EGFP fusion proteins in the HEK293 cells. EGFP-tagged PSAP; p.N157S displayed a lower co-localization with perinuclear lysosomal LAMP1-positive puncta compared to wild-type PSAP (Fig. 3C, D). The reduced lysosomal localization of the p.N157S variant may be due to an overall reduction in protein levels 48 h post-transfection (Fig. 3D, top panel), indicating that variant PSAP protein is less stable than that of wild-type protein in HEK293 cells. It has previously been shown that the intersaposin A–B region in the PSAP protein is necessary for cleavage of native PSAP into biologically active Saposin peptides^26^ (Fig. 3A, Saposin A–D). PSAP is a functional partner of lysosomal Cathepsin D (CTSD) and both proteins are mutually dependent for lysosomal trafficking. Moreover, PSAP–CTSD interaction is necessary for stimulation of the CTSD proteolytic activity, which mediates the processing of PSAP at the intersaposin A–B region^26–29^. Computational predictions indicated that p.N157S substitution in PSAP introduces a novel phosphorylation site (LESSKI, AA 153–158) in the intersaposin A–B region near a predicted CTSD-specific site (PSAP residues 176–179, LLL) (Fig. 3A, B and Supplementary Fig. 3A). We tested the influence of p.N157S substitution on the PSAP–CTSD interaction. Fluorescence resonance energy transfer (FRET) analysis of co-expressed PSAP and CTSD as FRET capable fusion proteins indicated that the p.N157S substitution reduces PSAP–CTSD interaction in the LAMP1+lysosomal perinuclear compartment (Supplementary Fig. 3B). Thus, reduced PSAP synthesis and reduced interaction with CTSD were identified as functional consequences of the p.N157S substitution. This may influence cellular alpha-synuclein homeostasis^30–33^ considering the role of PSAP in lysosomal CTSD delivery and activation^27,28^. We, therefore, tested the influence of the PSAP; p.N157S expression on lysosomal handling of alpha-synuclein in heterologous cells. To this end, we co-expressed mCherry-tagged PSAP or PSAP; p.N157S with an aggregation-prone alpha-synuclein-EGFP (see “Methods”). The number of LAMP1-positive alpha-synuclein-EGFP decorated inclusions per cell as well as the number of cells with such inclusions was higher when co-expressing PSAP; p.N157S than when co-expressing wild-type PSAP (Fig. 3E, F). Collectively, these results indicate that expression of p.N157S variant PSAP in HEK293 cells results in a lysosomal defect, which affects alpha-synuclein handling.

## Discussion

We here report rare genetic variants in the HSP70 chaperone DNAJB6 and the lysosomal protein PSAP as likely second hits in addition to the *GBA1* gene variants in two PD families. The segregation pattern of additional rare variants in both families (Fig. 1A, C) and their ability to manipulate cellular alpha-synuclein handling when expressed in reporter cells (Figs. 2C, D and 3E, F) lead to the hypothesis that they contribute to the PD pathophysiology.

The precise mechanism by which *GBA1* variants increase the risk for developing alpha synucleinopathy in PD remains unknown. Cellular alpha-synuclein is intrinsically prone to aggregation and toxic fibrillation^34,35^. Thus, a major portion of alpha-synuclein is delivered to lysosomes for degradation via non-vesicular (chaperone-mediated) or vesicular (micro- and macro-autophagy) pathways^36,37^. It was proposed that *GBA1* variants result in an abnormal lysosomal glycosphingolipid environment that leads to an impaired autophagy–lysosomal pathway and promotes alpha-synuclein aggregation^38^. However, genetic observations such as reduced PD penetrance^5^ of *GBA1* variants in familial cases, the occurrence of phenocopies in GBA-PD families^3,4^, and comparable age‐specific PD risk in homo-and heterozygous *GBA1* variant carriers^39^ indicate that *GBA1* variants alone do not cause alpha synucleinopathy or PD in the heterozygous *GBA1* variant carriers. This suggests that additional risk influence cellular alpha-synuclein homeostasis in GBA-PD.

DNAJB6 is a member of the HSP40 family of J-protein co-chaperones. Rare genetic variants in several members of this family have previously been associated with PD^40–44^. Our data support that a p.T193A substitution, which is located in a conserved serine-threonine-rich SxSTST motif (residues 190 and 192–195) of DNAJB6, affects its anti-aggregation properties with respect to cellar alpha-synuclein homeostasis as was described previously for other aggregation-prone proteins^19,20^. In our study, DNAJB6; p.T193A and *GBA1*; c.115+1G>A variants were found to segregate concurrently only in the PD patients of family A (Fig. 1A, B). Individuals lacking this concurrence did not show cardinal motor or prodromal signs of PD (Fig. 1A, subjects IV:6, IV:7, V:4, and V:5). The DNAJB6; p.T193A variant when expressed in HEK293 cells promotes alpha-synuclein aggregation (Fig. 2C, D). Taken together, our results indicate that DNAJB6; p.T193A is a rare genetic variant, which apparently acts independently in relation to GBA, to enhance PD risk in the *GBA1*; c.115+1G>A variant carriers of the family A.

PSAP is a multifunctional protein, which is secreted in its native form as ligand for the PD-associated neuroprotective receptor GPR37 (refs. ^45,46^). PSAP is intracellularly cleaved by lysosomal proteases such as Cathepsin D (CTSD)^26^ into mature monosaposins. One of the cleavage products, namely Saposin C, is essential cofactor for lysosomal GBA^24,25^. Short peptides derived from PSAP (e.g. Prosaptides) have protective effects on dopaminergic neurons in vitro and in models of PD in vivo^47,48^. At the molecular level, PSAP protein engages in reciprocal functional interaction with CTSD and this interaction affects lysosomal trafficking and function of both PSAP and CTSD^27–29^. Interestingly, *PSAP* mRNA is upregulated in the substantia nigra of PD patients^49^. This upregulation may be a protective mechanism against PD, given the neuroprotective functions of PSAP and our study, which indicates that a dysfunctional PSAP contributes to PD.

In family B, three PD patients were concurrent carriers of GBA; p.L444P and PSAP; p.N157S variants. However, patient III:4 carried only the PSAP; p.N157S variant. This patient was clinically indistinguishable from the three PD patients with both variants, indicating a similar underlying pathogenic mechanism (Supplementary Table 1, Family B). Indeed both GBA and PSAP proteins interact at the molecular level^24,25^. PD pathogenesis due to GBA; p.L444P variant involves accumulation of alpha-synuclein aggregates^50,51^. The PSAP; p.N157S variant when expressed in HEK293 cells also promoted alpha-synuclein aggregation (Fig. 3E, F). The exact molecular mechanism by which PSAP; p.N157S variant promotes alpha-synuclein is currently not known. One possibility is a reduced molecular interaction between PSAP; p.N157S variant and CTSD (Supplementary Fig. 3B), which may decrease lysosomal CTSD function^27^. Since CTSD is the main lysosomal protease clearing alpha-synuclein^52,53^, the PSAP; p.N157S variant may promote alpha-synuclein aggregation and PD risk independently of GBA; p.L444P variant. Thus, the PSAP; p.N157S variant may contribute to PD risk in all four PD patients of the family B independent of the GBA; p.L444P variant. We thus considered the subject III:4 of the family B to be a genetic phenocopy lacking the GBA; p.L444P variant.

Finally, we focused on rare genetic variation as additional risk factors for GBA-PD in a family-based design. Variants in the *PSAP* and *DNAJB6* genes were selected from the list of background WGS candidates primarily based on segregation testing and the potential roles of DNAJB6 and PSAP proteins in the cellular alpha-synuclein handling. In this study, we tested in heterologous cells (HEK293 cells) whether the identified variants have disrupted functions compared to the wild-type DNAJB6 and PSAP proteins. Further investigation on patient-derived material such as iPSCs is required to definitely link the identified rare genetic variants in DNAJB6 and PSAP with PD pathogenesis. The prioritization of DNAJB6 and PSAP in this study does not ignore the role of other rare variants in the list of candidates (Table 1) that may in addition play a role in PD pathogenesis. For instance, a potential pathogenic rare variant in the Tenascin receptor gene (*TNR*: p.N180H; ClinVAR ID 224863) was found in family A (Table 1). This finding is an independent replication of a recent exome sequencing study involving familial PD patients from a distant population^54^. In our study, the *TNR* variant was detected in three of the four PD patients of family A but in none of the controls, which indicates that the variant may have a pathogenic effect in the segregating individuals. In addition, we did not consider common genetic variation at the loci associated with cellular alpha-synuclein homeostasis. Large-scale genetic association studies are required to uncover such interactions. Despite these limitations, findings from our study contribute to a deeper molecular understanding of GBA-PD. Such understanding will not only be helpful for the clinicians when providing genetic counseling to seemingly GBA-PD patients and families but also for selecting patients for novel interventional therapies aiming to treat PD by augmenting lysosomal GBA function.

## Methods

### Study participants and clinical evaluation

Our study followed the tenets of the Declaration of Helsinki. The Institutional Review Board of the Institute for biomedical and genetic engineering (IBGE, Islamabad, Pakistan) provided the approval (Ref. IBGE/SARK04/1201/2012). Written informed consent was obtained from all participants.

Medical records of the neurology department of Shaheed Zulfiqar Ali Bhutto Medical University in Islamabad were screened rigorously to identify PD patients with a positive family history (defined as having at least one first-degree relative with PD who was also registered in the same clinic). We identified two families (family A and B) with four PD patients each. There was no history of consanguinity in the two families. After obtaining written informed consent, members of family A and B underwent a detailed neurologic examination by at least one experienced movement disorders specialist. The diagnosis of the eight PD patients was verified based on the UK Parkinson’s disease society brain bank^55^ and Gelb criteria^56^. Clinical data were collected, which comprised assessment of motor disability scores (Unified Parkinson Disease Rating Motor subscale III, UPDRS-III, total score 108)^57^, PD severity assessment (Hoehn and Yahr scale, H-Y, stages 1–5)^58^, cognitive impairment rating (Mini-Mental State Examination, MMSE, cut-off 24/30)^59^, depression rating (7-item version of the Hamilton Depression Rating Scale; scores 0–8: normal, 9–16: mild, 17–24: moderate and ≥25: severe)^60^ and an assessment of olfactory and visual functions.

### Genome sequencing and analysis

Genomic DNA was isolated from peripheral blood cells using a QIAamp DNA Blood Mini Kit. DNA libraries were prepared using the TruSeq Nano DNA PCR-free kit (Illumina San Diego CA USA). Paired-end sequencing (2 × 150 bp) was performed at >30× coverage per sample (Illumina HiSeq X TEN and HiSeq2500, Illumina San Diego CA). Raw reads were aligned to the human reference genome (version build GRCh37, version hs37d5) using bwa mem (version 0.7.8) with minimum score threshold set to zero [-T 0] and remaining settings left at default values^61^. Duplicates were marked using biobambam (version 0.0.148)^62^. SNVs were called using SAMtools (version 0.1.19)^63^ and short indels were called using Platypus (version 0.7.4)^64^. Functional classifications of the variants were done using ANNOVAR^65^ with gene model definitions from Gencode (version v19).

### Variant filtering, candidate selection, and Sanger sequencing

Quality-filtered variants (QUAL score >20) with minimum coverage >10× were carried forward for filtering and candidate selection. A stringent filtering approach was used to prioritize rare pathogenic variants likely representing candidates worthy of functional validation. We only considered variants with minor allele frequency MAF < 0.01 in ExAC (version 0.3) since high-risk rare variants with large effect sizes tend to aggregate in families. All functional rare variants, including nonsynonymous SNVs, frameshift, and non-frameshift indels, stop-gain/loss and splice-site SNVs or indels were considered. Rare functional variants that were identified only in the control individuals but were absent in one or more of the affected individuals of the family were removed. Deleteriousness prediction was carried out using a set of prediction tools, including CADD^16^, PolyPhen-2 (ref. ^66^), SIFT^67^, LRT^68^, Mutation Taster^69^, FATHMM^70^, PROVEAN^71^, and MetaSVM and MetaLR^72^. Benign variants were filtered out using the mutation significance cut‐off (MSC) tool, a gene‐level‐specific cut‐off for CADD/PolyPhen-2/SIFT scores as described previously^73,74^. Variants in genes with high brain expression in the GTEx Portal^75^ were prioritized. The SmallPedigree-WGS module of the Canvas program (version 1.40.0.1613) (https://academic.oup.com/bioinformatics/article/32/15/2375/1743834) was applied to the WGS data of two families separately to detect CNVs.

Commercially available M13-tailed primer pairs (Table 1; Thermo Fisher Scientific) were used for the amplification of candidate genomic regions. The genomic segment covering the position of p.L444P variant in *GBA1* (rs421016 position) was amplified using forward (TGGGTGCGTAACTTTGTCGACAG) and reverse (CCACAGCAGGATCCTTGATGGTAA) primers, as described previously^76^. The genomic segment covering a novel stop-gain variant in the ABCA7 gene (corresponding to p.Q1062X) was amplified using M13-tailed forward (TACTACCTGACGCTGGTGAAGG) and reverse (AGTTAAGGCACAGCCACCCCACTG) primers (M13 sequence not shown). Thermocycling conditions used were as follows: 3 min at 94 °C, 30 cycles of 94 °C for 30 s, 60 °C for 30 s, and 72 °C for 45 s, and a final extension for 5 min at 72 °C. PCR products were resolved on 1.5% agarose gel and purified using the MinElute Gel Extraction Kit (Qiagen, Germany). Bidirectional Sanger sequencing was performed at StarSEQ GmbH, Mainz, Germany.

### In silico analysis and protein structure predictions

Online tools PROSITE My Domains^77^, ClustalOmega^78^, and Jalview^79^ were used for drawing protein domain features, sequence alignment, and visualization, respectively. N-glycosylation sites were predicted using the GlycoEP and NetNGlyc web-servers^80,81^. Analysis of post-translational modification sites was performed with the Eukaryotic Linear Motif web-server^82^. Three-dimensional structure of human PSAP protein (Uniprot ID: P07602) was predicted using the I-TASSER web-server^83^. The ModLoop web-server^84^ was used to repair broken peptide bonds. Previously, Sun et al.^85^ showed that Cathepsin D (CTSD) specific sites are leucine rich. Based on this observation, we scanned the PSAP sequence, which predicted residues 176–179 (LLL) in the intersaposin region A–B of the PSAP as a potential CTSD-specific site. The three-dimensional structure of the human DNAJB6 homodimer was described previously by Söderberg et al.^19^ and was kindly provided by Professor Cecilia Emanuelsson (Lund University, Sweden). Figures of the PSAP and DNAJB6 structures were generated with PyMOL Molecular Graphics System (version 1.8).

### Plasmids, cell culture, and transfection

Full-length cDNA encoding wild-type human *PSAP* cloned in pCMV3 with a C-terminal Flag tag was obtained from Sino Biological (HG16224-CF) and subcloned into pEGFP-N1 with *Hin*dIII and *Age*I to obtain pEGFP-N1-PSAP (WT) plasmid. A fragment of *PSAP* cDNA encoding PSAP amino acid residues 1–320 encoding N157S variant was synthesized via GeneArt synthesis (Thermo Fisher Scientific) and inserted in pEGFP-N1-PSAP (WT) via *Hin*dIII and *Eco*RI, replacing the corresponding wild-type sequence to generate the pEGFP-N1-PSAP (N157S) plasmid. PSAP cDNA sequences encoding the wild type and the p.N157S variant were similarly cloned in pmCherry-N1. The cDNA sequence encoding human Cathepsin D (CTSD) was amplified from a human brain cDNA library and inserted into pmCherry-N1 using *Hin*dIII and *Age*I restriction sites. The pcDNA3.1 plasmid-expressing wild-type DNAJB6 as a fluorescent fusion protein was previously published^86^ and was kindly provided by Professor Conrad Chris Weihl (Washington University School of Medicine). A wild-type *DNAJB6* cDNA clone was modified by PCR to introduce the c.A577G/p.T193A variant in the coding sequence using the following primers: CAAATCGATATCAGCTTCAAC (forward primer; variant position is underlined) and CAACAGATGGCTGGCAACTAG (reverse primer). An alpha-synuclein-dsRed aggregation reporter cell line lacking endogenous DNAJB6 (ref. ^21^) was kindly provided by Dr. Cristian Hansen, Lund University, Sweden. A bicistronic plasmid encoding aggregation-prone alpha-synuclein (A53T) cDNA tagged with PDZ-binding motif (HSTTRV) of neuroligin-1 and PDZ1 domain of synaptic scaffolding molecule (S-SCAM) fused to mCherry has previously been described^87^ and was kindly provided by Professor Björn H. Falkenburger (University Hospital Carl Gustav Carus at the Dresden University of Technology, Germany). This plasmid was modified to replace mCherry with the EGFP coding sequence. A plasmid encoding Lamp1-mTurquoise2 was obtained from Addgene (Plasmid # 98828). Sanger sequencing verified the sequence integrity of all the above-mentioned plasmids.

Human embryonic kidney 293 cells (HEK293) were cultured in high glucose Dulbecco’s modified Eagle medium supplemented with 10% fetal bovine serum (Gibco) and 1% penicillin–streptomycin (Gibco). For transient transfections, HEK293 cells were grown on circular glass coverslips (13 mm diameter) coated before for 1 h with 300 μl of 0.1% (w/v) poly-l-lysine solution (Sigma). Cells were transfected with plasmid DNA (0.25 µg each) using Lipofectamine 2000 reagent in OPTIMEM medium (Thermo Fisher Scientific).

### Immunoblotting, cell imaging, and quantification

For immunoblotting, HEK293 cells transfected with plasmids expressing Flag-tagged proteins were washed and lysed. SDS-PAGE was performed with 10% acrylamide gels. Proteins were transferred to polyvinylidene difluoride (PVDF) membrane, which was blocked in 10% skimmed milk for 1 h. The membranes were incubated with mouse anti-Flag antibody (1:1000, F1804, Sigma) overnight at 4 °C, followed by incubation with peroxidase-linked secondary antibody (1:10,000, P0161, Dako) for 1 h. Protein bands were detected on a ChemiDoc XRS + imaging system (Bio-Rad). After Flag tag detection, the PVDF membrane was stripped and beta-actin expression was detected on the same membrane using a rabbit anti-actin antibody (1:1000, ab8227, Abcam).

For cell imaging, cells transfected with appropriate plasmids were washed three times with phosphate buffer saline (PBS) solution and were fixed in 4% paraformaldehyde for 10 min. Nuclei were stained with 4′,6′-diamidino-2-phenylindole (Sigma, St Louis, MO, USA). Coverslips were mounted onto slides with ProLong Gold anti-fade mountant (Thermo Fisher Scientific). Images were obtained with a ×63/1.4 oil objective on a TCS SP5 confocal imaging system (Leica Microsystems, Heidelberg GmbH). For the analysis of lysosomal localization of PSAP variants, Fiji software was used to quantify the integrated intensity of EGFP fluorescence (i.e PSAP-EGFP fusion) overlapping with turquoise signal (i.e mTurquoise2-LAMP1 fusion). At least 30 cells for each condition from triplicate coverslips were analyzed. Quantification of alpha-synuclein aggregation was performed as described previously^21,87^. Briefly, whole-field images were obtained from three coverslips for each condition. Alpha-synuclein aggregation was defined as a cellular region with markedly increased corresponding immunofluorescence signal compared with surrounding cytoplasmic areas of the same cell. An investigator blinded to the experimental conditions classified at least 100 cells per coverslip as positive or negative for cytoplasmic alpha-synuclein aggregates (Fig. 2), or cells with no, single, or multiple lysosomal aggregates (Fig. 3). All experiments were repeated at least three times.

### FRET analysis

For the FRET experiments, cells were transfected with plasmids expressing EGFP and mCherry as a donor–acceptor FRET pair alone as controls or as fusion proteins. A construct expressing EGFP and mCherry as a tandem dimer separated by a spacer was used as FRET standard. Twenty-four hours after transfection, cells were fixed and mounted as described above. FRET measurements were performed by using the FRET acceptor photobleaching wizard in the Leica Application Suite on a Leica scanning confocal microscope (TCS SP5 Leica Microsystems, Heidelberg GmbH, Germany). FRET measurements were performed for at least three independent transfections for each condition. FRET efficiency was defined as FRET_eff_ = (donor_post_ − donor_pre_)/donor_post_, where donor_post_ describes the fluorescence intensity after, and donor_pre_ the intensity before acceptor photobleaching. FRET_eff_ was calculated by the FRET wizard of the Leica Application Suite.

### Statistical analysis

We used QtiPlot software (version 4.8.7) for statistical analysis and data presentation. Graphs represent mean ± standard deviation. The statistical tests and number of independent experiments for each analysis are noted in the figure legends. *P* < 0.05 was considered significant.

MAÖ and RCW gratefully acknowledge the support of the Klaus Tschira Foundation and the European Union’s Horizon 2020 Framework Programme for Research and Innovation under the Specific Grant Agreement Nos. 720270 and 785907 (Human Brain Project SGA1 and SGA2). A.U. and S.N. gratefully acknowledge the support from the German Academic Exchange Service (DAAD Scholarship) and International Research Support Initiative Program of the higher education commission of Pakistan respectively.

### Reporting summary

Further information on experimental design is available in the Nature Research Reporting Summary linked to this paper.

## Supplementary information

Supplementary Information

Reporting Summary

## Data Availability

The datasets generated during and/or analyzed during the current study are available in the EGA repository under the following accessions: Study: EGAS00001004777 and Dataset: EGAD00001006561.

## Code availability

The custom scripts used to annotate, parse, and filter the variants are made available in the following public repositories:

https://github.com/NagaComBio/PlatypusGermlinePipeline, https://github.com/NagaComBio/GermlineSmallVariantAnalysis.

Following tools were used to map, de-duplicate, and call variants from the sample: bwa mem (version 0.7.8, parameter: -T 0), biobambam (version 0.0.148), SAMtools (version 0.1.19), Platypus (version 0.7.4). The following tools were used to annotate the variants: ANNOVAR, Gencode (version v19), CADD (version 1.3), dbNSFP (version 2.9). The germline CNVs were called used the SmallPedigree-WGS module of the Canvas program (version 1.40.0.1613).

The ClinVar accession numbers of the deposited variants are as follows: SCV001244288 and SCV001244287.
