# Supplementary material for: Putative second hit rare genetic variants in families with seemingly GBA-associated Parkinson’s disease
Source: NPJ Genom Med. 2021 Jan 5;6:2. doi: 10.1038/s41525-020-00163-8 (PMC7785741; doi:10.1038/s41525-020-00163-8)

Supplementary file.

Putative second hit rare genetic variants in families with seemingly GBA associated Parkinson's disease

Muhammad Aslam[a], Nirosiya Kandasamy[a], Anwar Ullah[a,b,c], Nagarajan Paramasivam[d], Mehmet Ali Öztürk[e,f], Saima Naureen[a,g], Abida Arshad[g], Mazhar Badshah[h], Kafaitullah Khan[i], Muhammad Wajid[j], Rashda Abbasi[b], Muhammad Ilyas[k], Roland Eils[l,m], Matthias Schlesner[n], Rebecca C. Wade[e,o], Nafees Ahmad[b], Jakob von Engelhardt[a]

Supplementary table 1. Demographic and clinical characteristics of PD patients in family A and B.

Family A

Case ID	Onset (years)	Duration (years)	UPDRS motor examination	H&Y stage	MMSE score	Depression (HAM-D)
IV:1	51	18	44/108	4	18	Severe
IV:4	48	15	38/108	3	20	Severe
IV:5	47	12	30/108	3	28	Mild
V:1	40	5	24/108	2	30	Moderate

Family B

Case ID	Onset (years)	Duration (years)	UPDRS motor examination	H&Y stage	MMSE score	Depression (HAM-D)
II:2	59	21	88/108	5	26	No
II:4	54	17	54/108	3	12	Severe
III:1	42	10	36/108	2	26	Severe
III:4	51	4	14/108	1	30	No

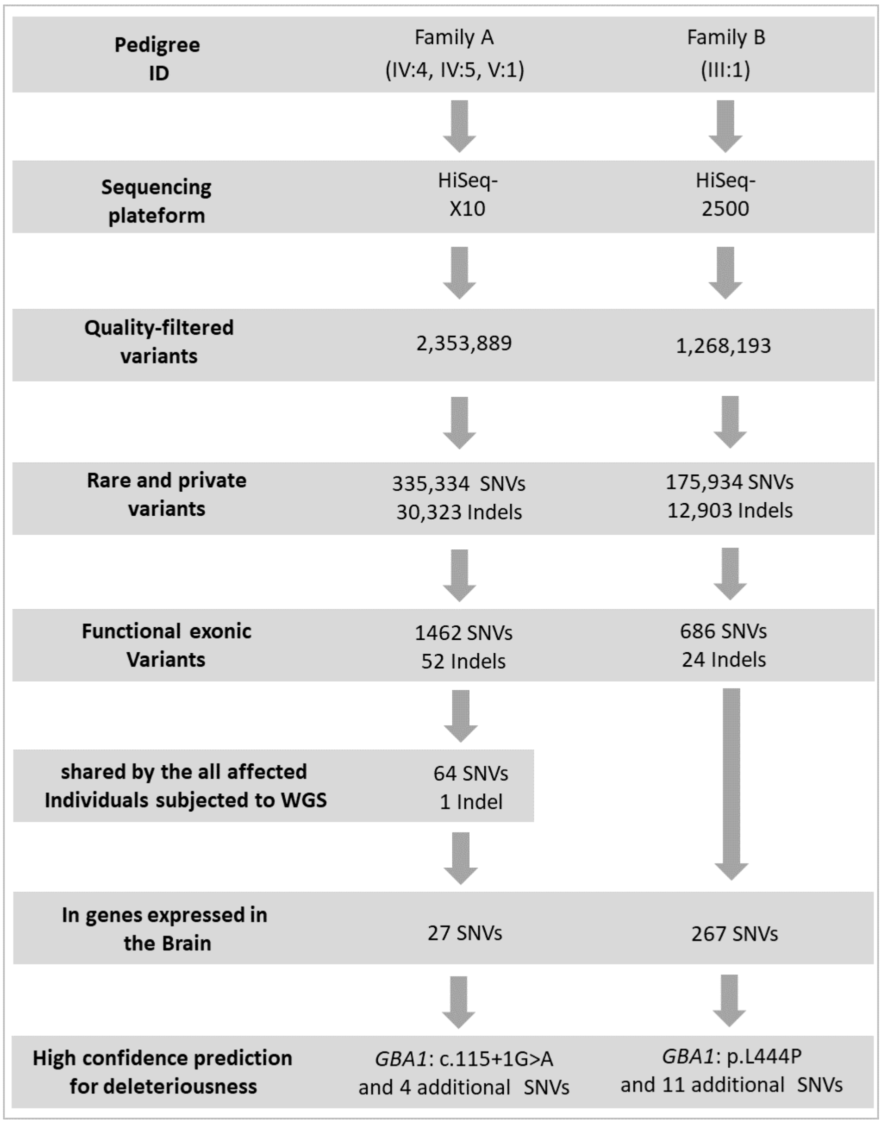

Supplementary figure 1. Study design and variant filtering

Shown is the overall design for the study and variant counts at each stage of filtering. Indels, insertions or deletions; MAF, minor allele frequency; and SNVs, single-nucleotide variants.

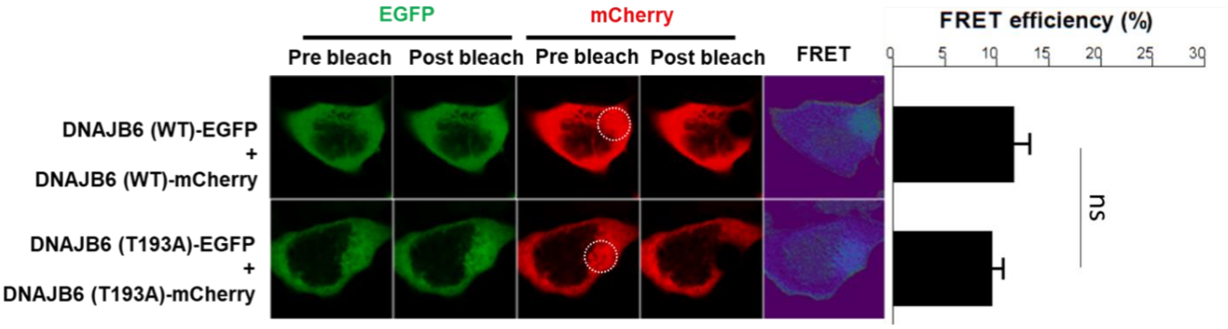

Supplementary figure 2. Assessment of DNAJB6 dimerization by fluorescence resonance energy transfer (FRET) measurement with acceptor photobleaching in HEK-293 cells.

Wildtype or T193A variant containing DNAJB6 was expressed as EGFP and mCherry fusion proteins (FRET donor and acceptor, respectively) in HEK293 cells. Representative images of separate channels before and after acceptor photobleaching are shown; white circles in the mCherry (acceptor channel) mark regions of photobleaching. The donor (EGFP-DNAJB6) fluorescence increases when the acceptor (mCherry-DNAJB6) fluorescence is subjected to photobleaching. Data were collected from 20 cells per condition. Quantification of the FRET signal is also shown (bar graph). Statistical analysis was performed by t-test, Means ± S.D. n.s. $p > 0.05$.

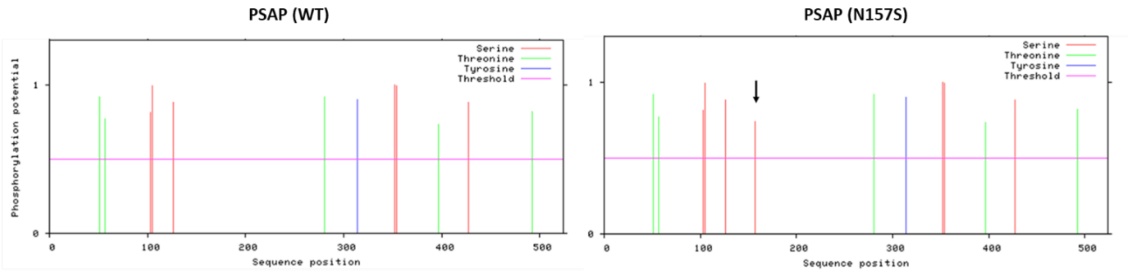

Supplementary figure 3A. Prediction of post-translational modifications.

NetPhos 3.1 Server output showing prediction results for phosphorylation sites in wildtype and p.N157S variant containing PSAP. An arrow indicates the novel phosphorylation site in the p.N157S variant containing PSAP.

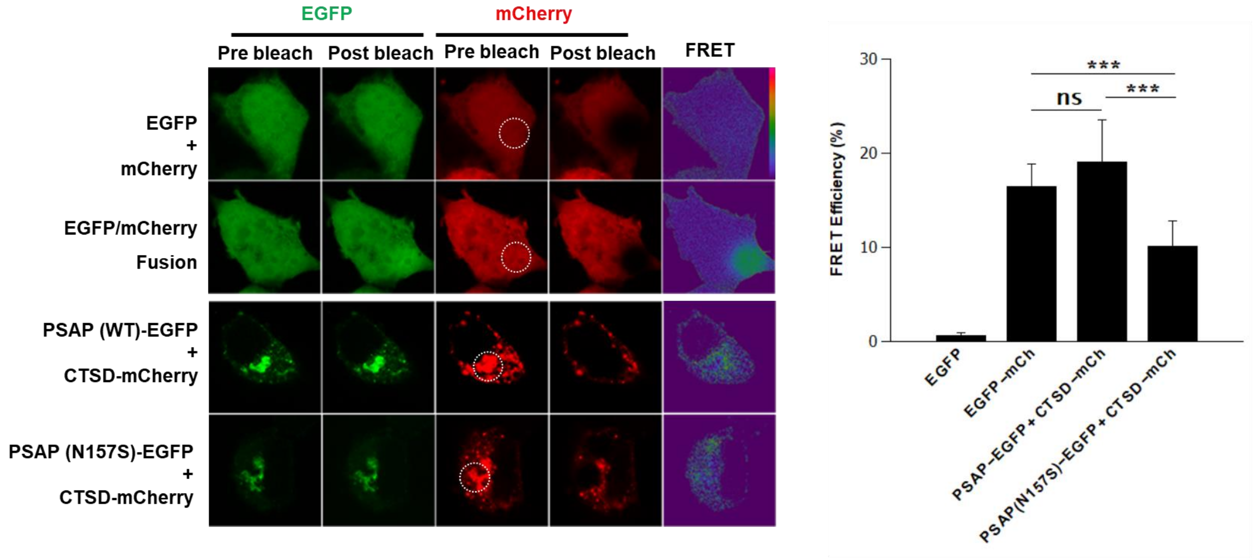

Supplementary figure -3B. Assessment of PSAP and Cathepsin D (CTSD) interaction by fluorescence resonance energy transfer (FRET) measurement with acceptor photobleaching in HEK-293 cells.

Cells were co-transfected with plasmids expressing wildtype or p.N157S variant containing human PSAP as EGFP fusion protein and human Cathepsin D (CTSD) precursor as mCherry fusion protein in HEK293 cells as FRET donor-acceptor pair respectively. As a control, cells were transfected with EGFP and mCherry separately or as a FRET capable conjugate. Representative images of separate channels before and after photobleaching are shown; white circles in the mCherry channel (FRET acceptor) mark regions of photobleaching. The donor fluorescence (EGFP-PSAP fusion) increases when the acceptor fluorescence (mCherry-CTSD fusion) is subjected to photobleaching. Data were collected from 20 cells per condition. Quantification of the FRET signal is shown in the right panel. Statistical analysis was performed by ANOVA. ***$P < 0.001$, n.s. $p > 0.05$.

Supplementary figure -3C. Images of original uncropped Western blots used for preparation of Figure 3D.

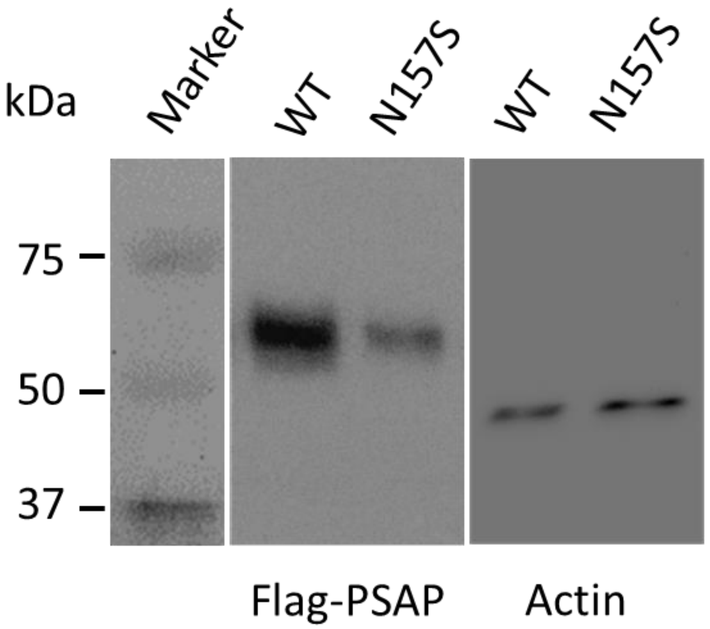

Supplement: Supplementary file 1 — Supplementary Information [file 41525_2020_163_MOESM1_ESM.pdf]
