# Supplementary material for: Putative second hit rare genetic variants in families with seemingly GBA-associated Parkinson’s disease
Source: NPJ Genom Med. 2021 Jan 5;6:2. doi: 10.1038/s41525-020-00163-8 (PMC7785741; doi:10.1038/s41525-020-00163-8)
Supplement: Supplementary file 2 — Reporting Summary [file 41525_2020_163_MOESM2_ESM.pdf]

nature research

Corresponding author(s): Muhammad Aslam
Jakob von Engelhardt

Last updated by author(s): Jun 24, 2020

Reporting Summary

Nature Research wishes to improve the reproducibility of the work that we publish. This form provides structure for consistency and transparency in reporting. For further information on Nature Research policies, see our Editorial Policies and the Editorial Policy Checklist.

Statistics

For all statistical analyses, confirm that the following items are present in the figure legend, table legend, main text, or Methods section.

n/a	Confirmed	
☐	☒	The exact sample size (*n*) for each experimental group/condition, given as a discrete number and unit of measurement
☐	☒	A statement on whether measurements were taken from distinct samples or whether the same sample was measured repeatedly
☐	☒	The statistical test(s) used AND whether they are one- or two-sided<br>*Only common tests should be described solely by name; describe more complex techniques in the Methods section.*
☒	☐	A description of all covariates tested
☒	☐	A description of any assumptions or corrections, such as tests of normality and adjustment for multiple comparisons
☒	☐	A full description of the statistical parameters including central tendency (e.g. means) or other basic estimates (e.g. regression coefficient) AND variation (e.g. standard deviation) or associated estimates of uncertainty (e.g. confidence intervals)
☐	☒	For null hypothesis testing, the test statistic (e.g. *F*, *t*, *r*) with confidence intervals, effect sizes, degrees of freedom and *P* value noted<br>*Give P values as exact values whenever suitable.*
☒	☐	For Bayesian analysis, information on the choice of priors and Markov chain Monte Carlo settings
☒	☐	For hierarchical and complex designs, identification of the appropriate level for tests and full reporting of outcomes
☒	☐	Estimates of effect sizes (e.g. Cohen's *d*, Pearson's *r*), indicating how they were calculated

Our web collection on statistics for biologists contains articles on many of the points above.

Software and code

Policy information about availability of computer code

Data collection	The custom scripts were used to annotate, parse and filter the variants and they were made available in the following public repositories: https://github.com/NagaComBio/PlatypusGermlinePipeline, https://github.com/NagaComBio/GermlineSmallVariantAnalysis.
Data analysis	The following tools were used to map, de-duplicate and call variants from the sample: bwa mem (version 0.7.8, parameter: -T 0), biobambam (version 0.0.148), SAMtools (version 0.1.19), Platypus (version 0.7.4). The following tools were used to annotate the variants: ANNOVAR, Gencode (version v19), CADD (version 1.3), dbNSFP(version 2.9). The germline CNVs were called used the SmallPedigree-WGS module of the Canvas program (version 1.40.0.1613).

For manuscripts utilizing custom algorithms or software that are central to the research but not yet described in published literature, software must be made available to editors and reviewers. We strongly encourage code deposition in a community repository (e.g. GitHub). See the Nature Research guidelines for submitting code & software for further information.

Data

Policy information about availability of data

All manuscripts must include a data availability statement. This statement should provide the following information, where applicable:

- Accession codes, unique identifiers, or web links for publicly available datasets
- A list of figures that have associated raw data
- A description of any restrictions on data availability

Raw data were generated at Genomic facility of the German Cancer research Institute, Heidelberg, Germany. Analyzed data supporting the findings of this study are available from the corresponding author on request.

April 2020

Field-specific reporting

Please select the one below that is the best fit for your research. If you are not sure, read the appropriate sections before making your selection.

☒ Life sciences ☐ Behavioural & social sciences ☐ Ecological, evolutionary & environmental sciences

For a reference copy of the document with all sections, see nature.com/documents/nr-reporting-summary-flat.pdf

Life sciences study design

All studies must disclose on these points even when the disclosure is negative.

Sample size	Not applicable
Data exclusions	Not applicable
Replication	Not applicable
Randomization	Not applicable
Blinding	Experimenters were blinded to the experimental groups for the data shown in Figure 2 and 3.

Reporting for specific materials, systems and methods

We require information from authors about some types of materials, experimental systems and methods used in many studies. Here, indicate whether each material, system or method listed is relevant to your study. If you are not sure if a list item applies to your research, read the appropriate section before selecting a response.

Materials & experimental systems

n/a	Involved in the study	
☐	☒	Antibodies
☐	☒	Eukaryotic cell lines
☒	☐	Palaeontology and archaeology
☒	☐	Animals and other organisms
☐	☒	Human research participants
☒	☐	Clinical data
☒	☐	Dual use research of concern

Methods

n/a	Involved in the study	
☒	☐	ChIP-seq
☒	☐	Flow cytometry
☒	☐	MRI-based neuroimaging

Antibodies

Antibodies used	mouse monoclonal anti-Flag M2 (Sigma), Anti-beta Actin antibody produced in rabbit (Abcam).
Validation	Sigma Aldrich product number F1804, Abcam product number ab8227

Eukaryotic cell lines

Policy information about cell lines

Cell line source(s)	HEK293, ATCC
Authentication	www.atcc.org
Mycoplasma contamination	All cell lines were tested negative for mycoplasma contamination
Commonly misidentified lines (See ICLAC register)	not applicable

Human research participants

Policy information about studies involving human research participants

Population characteristics	not applicable

Recruitment	Medical records of the neurology department of Shaheed Zulfiqar Ali Bhutto Medical University in Islamabad, Pakistan
Ethics oversight	Institutional Review Board of the Institute for biomedical and genetic engineering (IBGE, Islamabad, Pakistan) (Ref. IBGE/SARK04/1201/2012).

Note that full information on the approval of the study protocol must also be provided in the manuscript.
